# Versatile Self-Assembly of Triblock Peptides into Stable Collagen Mimetic Heterotrimers

**DOI:** 10.3390/ijms25126550

**Published:** 2024-06-14

**Authors:** Linyan Yao, Biyang Ling, Sha Zhao, Fansen Yu, Huanxiang Liu, Shenlin Wang, Jianxi Xiao

**Affiliations:** 1 State Key Laboratory of Applied Organic Chemistry, College of Chemistry and Chemical Engineering, Lanzhou University, Lanzhou 730000, China; 2 School of Life Science, Lanzhou University, Lanzhou 730000, China; 3 College of Chemistry and Molecular Engineering, Beijing NMR Center, Peking University, Beijing 100871, China; 4 School of Pharmacy, Lanzhou University, Lanzhou 730000, China

**Keywords:** triblock peptides, heterogeneous proteins, heterotrimer, collagen, self-assembly

## Abstract

The construction of peptides to mimic heterogeneous proteins such as type I collagen plays a pivotal role in deciphering their function and pathogenesis. However, progress in the field has been severely hampered by the lack of capability to create stable heterotrimers with desired functional sequences and without the effect of homotrimers. We have herein developed a set of triblock peptides that can assemble into collagen mimetic heterotrimers with desired amino acids and are free from the interference of homotrimers. The triblock peptides comprise a central collagen-like block and two oppositely charged N-/C-terminal blocks, which display inherent incompetency of homotrimer formation. The favorable electrostatic attraction between two paired triblock peptides with complementary terminal charged sequences promptly leads to stable heterotrimers with controlled chain composition. The independence of the collagen-like block from the two terminal blocks endows this system with the adaptability to incorporate desired amino acid sequences while maintaining the heterotrimer structure. The triblock peptides provide a versatile and robust tool to mimic the composition and function of heterotrimer collagen and may have great potential in the design of innovative peptides mimicking heterogeneous proteins.

## 1. Introduction

The biomimicry of heterogeneous proteins composed of multiple peptide chains is of great significance for deciphering the molecular mechanism of complicated biosystems and developing multifunctional biomaterials [1,2,3,4]. The diverse chain composition of heterogeneous proteins plays a crucial role in modulating their physiological and pathological processes [4,5,6,7,8,9]. Type I collagen, the major structural component of extracellular matrix, is an exemplification of heterogeneous protein consisting of two identical α1 chains and one α2 chain, each comprising the repeating (Gly-X-Y)n triplets [10,11,12,13,14,15]. The heterotrimeric nature of type I collagen is essential for the fibrous morphology, mechanical strength and biological function of connective tissues; while its homotrimeric isoform tended to cause tumors, fibrosis, and other severe diseases [16,17,18]. Mimicking the heterotrimeric composition of type I collagen would greatly contribute to elucidating its function and pathogenesis [19,20].

Peptides have gained increasing attention in simulating type I collagen due to their advantages, including well-controlled sequences, suitable size, and convenient biophysical characterization [21,22]. A few innovative strategies have been developed to drive peptides consisting of the single (Gly-X-Y)n block to assemble into collagen heterotrimers, such as electrostatic interactions and computational optimization [23,24,25,26,27,28]. However, most of these approaches frequently depend on the presence of a homotrimer possessing a stable triple helical structure. Additionally, these previously reported heterotrimeric peptides necessitate complicated sequence design for the optimal positioning of specific amino acids, potentially lacking the versatility to readily incorporate novel functional motifs [27,28].

We have herein reported a versatile triblock peptide system that can assemble into collagen mimetic AAB heterotrimer with desired functional sequences and free from the interference of homotrimers. Two types of peptides consisting of a central collagen-like block and two N-/C-terminal blocks with oppositely charged amino acids have been created. Various desired amino acids, such as the integrin-binding sequences GFOGER and GPOGES, can be conveniently incorporated into the collagen-like block without affecting the formation of heterotrimer and successfully mimic the heterotrimeric peptide chain composition of native type I collagen. This novel heterotrimeric triblock peptide system provides a versatile and robust strategy to mimic the composition and structure of type I collagen and lays a foundation for the construction of self-assembled heterotrimer peptides.

## 2. Results

### 2.1. Rational Design of Triblock Peptides

**Figure 1:**
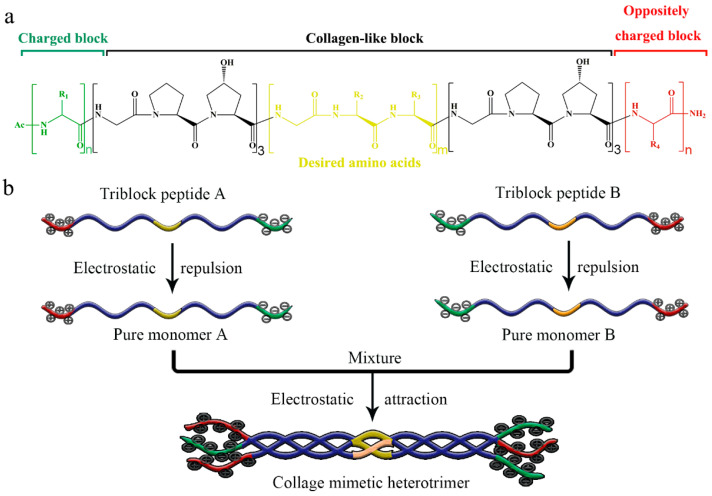
Schematic illustration of triblock peptides to form heterotrimer. Amino acid sequences of triblock peptides (**a**). The assembly of triblock peptides into heterotrimer with various amino acids and without the interference of homotrimers (**b**).

Herein, a set of triblock peptides has been constructed that can assemble into stable collagen mimetic heterotrimers without the interference of homotrimers. The triblock peptides are composed of three blocks: a central collagen-like block and two oppositely charged N- and C-terminal blocks (Figure 1a). We hypothesize that the electrical repulsion between the same charged amino acids would prevent the formation of a homotrimer; while mixing two oppositely charged triblock peptides would drive the formation of a stable heterotrimer due to the electrical attraction (Figure 1b). Notably, the central collagen-like block is independent, and various amino acids (Figure 1b, yellow marked area) can be easily incorporated without interfering with the fabrication of heterotrimer.

Two pairs of peptides: K-α1-D (with the sequence K_5_(GPO)_3_GFOGER(GPO)_3_D_5_) and D-α1-K (with the sequence D_5_(GPO)_3_GFOGER(GPO)_3_K_5_) as well as K-α2-D (with the sequence K_5_(GPO)_3_GPOGES(GPO)_3_D_5_) and D-α2-K (with the sequence D_5_(GPO)_3_GPOGES(GPO)_3_K_5_) have been constructed to demonstrate the robustness of the triblock peptide strategy to create AAB heterotrimers (Table 1). GFOGER is the integrin binding motif in the α1 chain of type I collagen, while GPOGES is the corresponding sequence in the α2 chain [13,29,30,31]. The recombined pairs of peptides K-α1-D and D-α2-K as well as K-α2-D and D-α1-K are examined to further illustrate the versatility of the triblock peptides to mimic the natural composition of type I collagen. Gly18 in peptides K-α1-D and D-α2-K is selectively ^15^N labeled, named as K-α1*-D and D-α2*-K, respectively. Peptides α1 (with the sequence (GPO)_3_GFOGER(GPO)_3_) and α2 (with the sequence (GPO)_3_GPOGES(GPO)_3_) are designed as control peptides (Table 1).

### 2.2. Stability of Homotrimers

**Figure 2:**
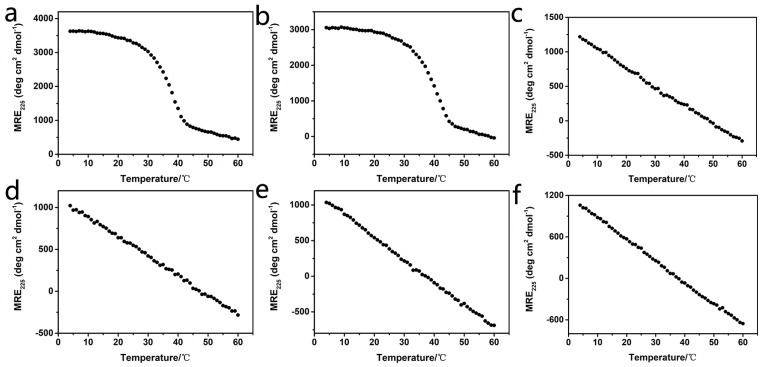
CD thermal unfolding curves of peptides α1 (**a**), α2 (**b**), K-α1-D (**c**), D-α1-K (**d**), K-α2-D (**e**) and D-α2-K (**f**).

The circular dichroism (CD) spectra of all triblock peptides have been recorded to investigate their triple-helical structure and thermal stability (Figure 2). Peptide solutions with a concentration of 0.45 mM in 10 mM phosphate buffer (pH 7.0) were used for the CD experiments. Peptides α1 and α2 exhibited a typical peak at 225 nm, indicating the formation of a triple helix (Figure S1). Thermal unfolding curves further showed that peptides α1 and α2 formed a stable triple helix with melting temperatures (T_m_) of 38.0 °C and 41.0 °C, respectively (Figure 2a,b). In contrast, the thermal unfolding studies of peptides K-α1-D, D-α1-K, K-α2-D, and D-α2-K displayed a linear decrease of the CD_225nm_ intensity during their melting, suggesting that all these triblock peptides could not form stable triple helix (Figure 2c–f). These results demonstrated that the inclusion of terminal-charged amino acids completely prevented the formation of homotrimers due to the electrical repulsion of the triblock peptides, which is consistent with our hypothesis.

### 2.3. Stability of Heterotrimers

**Figure 3:**
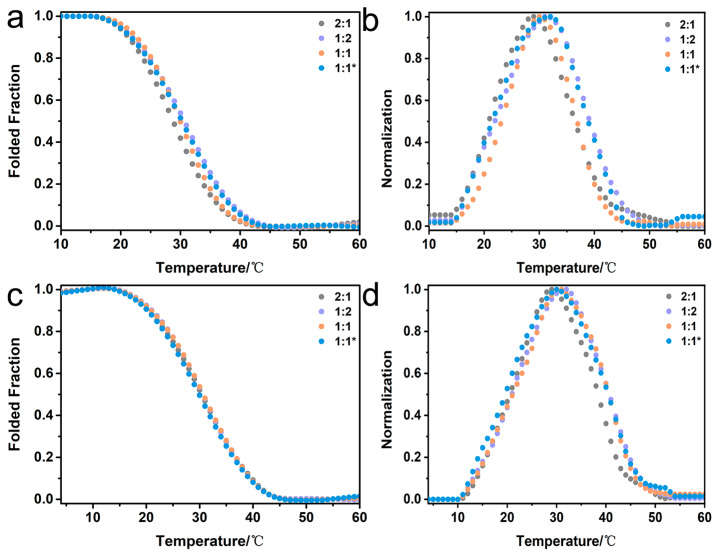
CD characterization of the mixtures of paired triblock peptides K-α1-D and D-α1-K (**a**,**b**) as well as K-α2-D and D-α2-K (**c**,**d**). Folded fractions of thermal unfolding curves monitored at 225 nm (**a**,**c**) and their corresponding first derivative (**b**,**d**). The peptide mixtures were prepared at different ratios of 2:1 (gray), 1:2 (purple), and 1:1 (orange) after preheating, and the 1:1 peptide mixture was also produced without preheating (marked with an asterisk; blue).

The CD spectra of peptide mixtures K-α1-D and D-α1-K, as well as K-α2-D and D-α2-K at different ratios of 2:1, 1:2, and 1:1, were carried out to look for the formation of heterotrimers (Figure 3). The peptide mixtures were preheated at 85 °C for 20 min and then incubated at 4 °C for 24 h. In order to compare the thermal variation of different heterotrimers more clearly, the absolute value of the first derivative has been normalized. Thermal unfolding studies of the K-α1-D and D-α1-K mixtures at different ratios indicated the formation of a stable triple helix with T_m_ of 29.0 °C (2:1, gray), 31.0 °C (1:2, purple) and 30.0 °C (1:1, orange), respectively (Figure 3a,b and Table S1). Similarly, the mixtures of K-α2-D and D-α2-K at different ratios displayed the formation of a triple helix in their unfolding curves with T_m_ of 30.0 °C (2:1, gray), 31.0 °C (1:2, purple), and 30.0 °C (1:1, orange), respectively (Figure 3c,d and Table S1). These results consistently demonstrated that the electrical attraction between two oppositely charged triblock peptides could trigger the formation of stable heterotrimers.

In order to evaluate the effect of preheating on the heterotrimer formation, the 1:1 mixtures of paired peptides K-α1-D and D-α1-K, as well as K-α2-D and D-α2-K were prepared at 4 °C, and their thermal unfolding curves were immediately measured (Figure 3, blue). The two un-preheated mixtures displayed the formation of a stable triple helix with T_m_ of 31.0 °C (Figure 3a,b, blue) and 30.0 °C (Figure 3c,d, blue), which were similar to the Tm of the corresponding preheated mixtures. It indicated that the two oppositely charged triblock peptides could readily form stable heterotrimers in a few minutes without any preheating, demonstrating the robustness of the triblock peptides’ strategy for the production of heterotrimers.

### 2.4. Mimicking the Natural Composition of Type I Collagen

**Figure 4:**
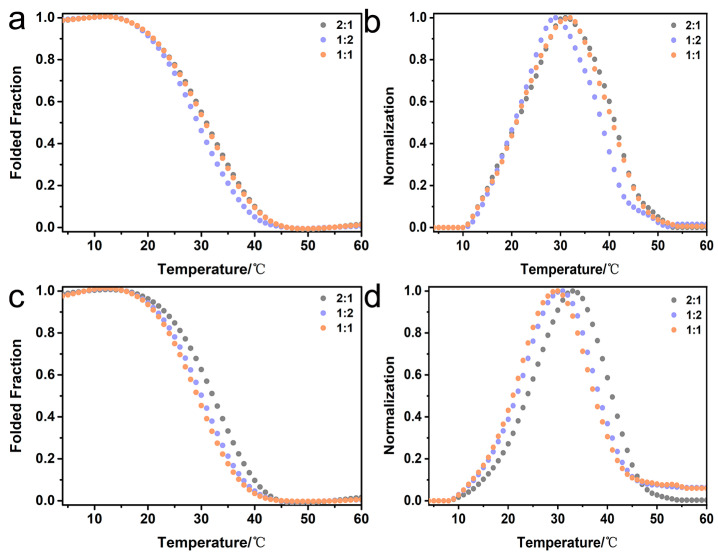
CD characterization of the mixtures of triblock peptides mimicking the natural composition of type I collagen. Folded fractions of thermal unfolding curves monitored at 225 nm (**a**,**c**), and the first derivative (**b**,**d**) of the peptide mixtures of K-α1-D and D-α2-K (**a**,**b**) as well as K-α2-D and D-α1-K (**c**,**d**). The peptide mixtures were prepared at different ratios: 2:1 (gray), 1:2 (purple) and 1:1 (orange).

In order to construct heterotrimers imitating the natural composition of type I collagen, the reconstituted pairs of peptides K-α1-D and D-α2-K as well as K-α2-D and D-α1-K were further investigated. Mixtures of the paired peptides K-α1-D and D-α2-K, as well as K-α2-D and D-α1-K, were prepared at molar ratios of 2:1, 1:2, and 1:1, preheated at 85 °C for 20 min, and then incubated at 4 °C for 24 h. The thermal unfolding curves of peptide mixtures of K-α1-D and D-α2-K displayed the formation of heterotrimers with T_m_ of 31.0 °C (2:1, gray), 29.0 °C (1:2, purple) and 31.0 °C (1:1, orange), respectively (Figure 4a,b and Table S2). Peptide mixtures of K-α2-D and D-α1-K indicated the formation of heterotrimers with similar T_m_ values of 32.0 °C (2:1, gray), 31.0 °C (1:2, purple), and 30.0 °C (1:1, orange), respectively (Figure 4c,d and Table S2). These results consistently demonstrated that two paired triblock peptides are capable of incorporating different amino acid sequences without any disturbance in the heterotrimer formation, indicating their superior versatility to mimic two different chains α1 and α2 in type I collagen.

### 2.5. Composition Analysis of Collagen Mimetic Heterotrimers

**Figure 5:**
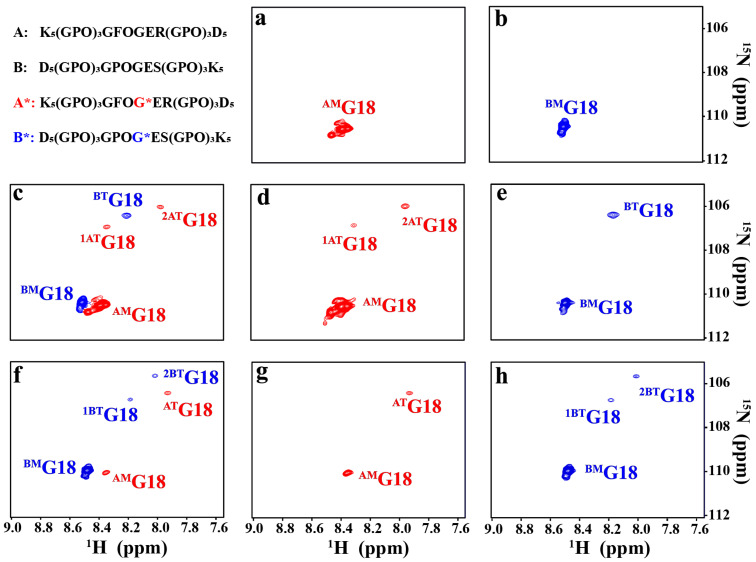
^1^H-^15^N HSQC spectra of peptides A* (**a**), B* (**b**), 2A*:1B* (**c**), 2A*:1B (**d**), 2A:1B* (**e**), 1A*:2B* (**f**), 1A*:2B (**g**) and 1A:2B* (**h**) in 10 mM phosphate buffer (pH 7.0) at 8 °C. Peptide sequences are shown at the upper left corner with ^15^N-labeled residues starred. The resonances corresponding to monomer and trimer states are denoted with superscripts M and T, respectively.

^1^H-^15^N heteronuclear single quantum coherence (HSQC) experiments were performed on two peptides K-α1*-D and D-α2*-K with ^15^N-labeled residue Gly18 to further examine their capability to form a heterotrimer. To simplify the description, peptides K-α1*-D, K-α1-D, D-α2*-K, and D-α2-K were renamed as peptides A*, A, B*, and B, respectively (Figure 5). The peaks corresponding to monomer and trimer states were denoted with superscript M and T, respectively, whereas its chain composition A and B was indicated as the superscript A and B, respectively. In the HSQC spectra of peptides A* and B* alone, only one monomer resonance for ^AM^G18 (Figure 5a) and ^BM^G18 (Figure 5b) was observed, respectively. It indicated that both peptides maintained the monomer conformation, which was consistent with their CD characterization. In contrast, the HSQC spectrum of the 2A*:1B* mixture displayed typical features for triple-helical conformation, with the ^15^N-labeled residue Gly18 showing three trimer resonances in addition to two monomer resonances (Figure 5c). The co-existence of monomer and trimer resonances indicated a trimer-monomer equilibrium in the 2A*:1B* solution. Two monomer resonances (^AM^G18 and ^BM^G18) were assigned to peptide chains A* and B*, respectively, since they overlapped with the monomer resonances in the HSQC spectra of peptides A* and B* alone.

In order to assign the trimer resonances, two peptide mixtures, 2A*:1B and 2A:1B*, were prepared. In the HSQC spectrum of 2A*:1B mixture, two trimer resonances (^1AT^G18 and ^2AT^G18) and one monomer resonance (^AM^G18) were observed (Figure 5d), while the HSQC spectrum of 2A:1B* mixture showed one trimer peak (^BT^G18) and one monomer peak (^BM^G18) (Figure 5e). By overlapping the HSQC spectra of 2A*:1B*, 2A*:1B, and 2A:1B*, the three trimer resonances for 2A*:1B* were assigned as ^1AT^G18, ^2AT^G18, and ^BT^G18, respectively. These results demonstrated that the 2A*:1B* mixture led to the formation of an A*A*B* heterotrimer composed of two A chains and one B chain.

The amide proton temperature gradients are indicative of hydrogen bonding, with a value higher than −4.6 ppb/°C demonstrating the presence of hydrogen bonds [32]. The monomer resonances ^AM^G18 and ^BM^G18 both showed much more negative values than the cut-off value, suggesting the absence of any hydrogen bonding. In contrast, the trimer resonances ^1AT^G18, ^2AT^G18, and ^BT^G18 displayed much larger values than −4.6 ppb/°C, supporting the formation of typical hydrogen bonds for Gly18 in all three chains in the heterotrimer (Figure S2). It indicated that the 2A*:1B* mixture assembled to form an A*A*B* heterotrimer with the conserved hydrogen bonding for Gly.

The 1A*:2B* mixture was also examined to investigate the effect of peptide ratios on the heterotrimer composition. The HSQC spectrum of the 1A*:2B* mixture displayed typical features for triple-helical conformation, with the ^15^N-labeled residue Gly18 showing three trimer resonances in addition to two monomer resonances, indicating a trimer-monomer equilibrium in solution (Figure 5f). Two monomer resonances ^AM^G18 and ^BM^G18 were assigned to peptide chains A*and B*, respectively, due to their overlap with the monomer resonances in the HSQC spectra of peptides A* and B* alone. Two peptide mixtures, 1A*:2B and 1A:2B*, were further investigated to facilitate the assignment of the three trimer resonances. In the HSQC spectrum of the 1A*:2B mixture, one trimer peak (^AT^G18) and one monomer peak (^AM^G18) were observed (Figure 5g), while the HSQC spectrum of the 1A:2B* mixture showed two trimer resonances (^1BT^G18 and ^2BT^G18) and one monomer resonance (^BM^G18) (Figure 5h). These results demonstrated that the 1A*:2B* mixture resulted in the formation of an A*B*B* heterotrimer comprising two A chains and one B chain. It is noteworthy that the heterotrimer composition depends on the relative concentration of peptides A and B in the mixture, while the peptide mixtures 1A:2B and 2A:1B leads to the formation of heterotrimer ABB and AAB, respectively.

### 2.6. Structural Features of Collagen Mimetic Heterotrimers

**Figure 6:**
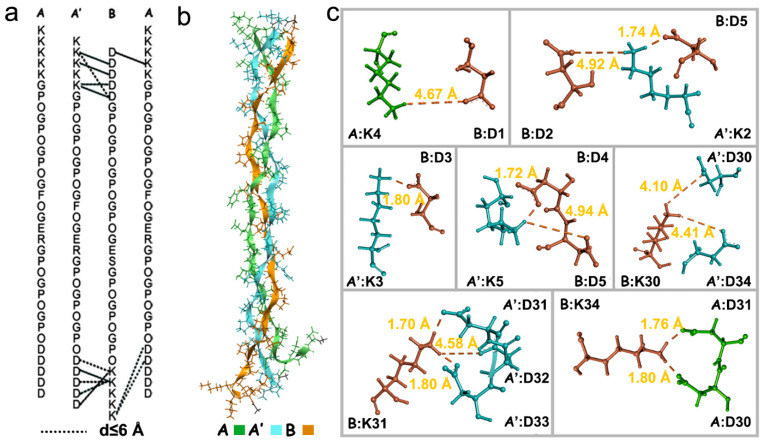
The structural features of collagen heterotrimer peptide AAB. (**a**) Sequence-based design. Salt bridges with distance d between Asp and Lys **d** < 6 Å are denoted by black dashed lines. (**b**) Model structure of peptide AA’B. (**c**) Salt-bridge distance distributions determined using Discovery Studio 4.5 Client software. ***A*** and ***A’*** were used to distinguish two peptide A chains.

A molecular dynamics (MD) simulation was performed for a timeframe of 600 ns to examine the structural features of heterotrimer peptide AAB (Figure 6). The RMSD graph depicted that the triple helix structure of AAB stabilized around 200 ns and thereafter remained stable till 600 ns with a final RMSD value of 5.9 Å (Figure S3a). The Rg graph showed an initial fluctuation and stabilized after 50 ns, suggesting the structure has formed a compact conformation (Figure S3b). The stable model structure of peptide AAB was used to investigate the salt bridge using Discovery Studio 4.5 Client software (Figure 6b,c). Thirteen salt bridges were identified between the oppositely charged amino acids K and D in the heterotrimer structure: ***A***:K4-***B***:D1, ***A’***:K2-***B***:D2, ***A***:K2-***B***:D5, ***A’***:K3-***B***:D3, ***A’***:K5-***B***:D4, ***A’***:K5-***B***:D5, ***B***:K30-***A’***:D30, ***B***:K30-***A’***:D34, ***B***:K31-***A’***:D31, ***B***:K31-***A’***:D31, ***B***:K31-***A’***:D32, ***B***:K34-***A***:D31, and ***B***:K34-***A***:D30 (***A*** and ***A’*** were used to distinguish two peptide A chains) (Figure 6c). These results suggested the formation of an extensive salt-bridge network between the oppositely charged terminal amino acids in the paired triblock peptides, resulting in enhanced stability of the heterotrimer AAB.

To further investigate the effect of electrostatic interaction on the heterotrimer formation, the thermal stability of 2A:1B mixtures at different pHs was further examined (Figure S4). CD thermal unfolding curves of the 2A:1B mixture at pH 3.0 (purple) and 11.0 (orange) both showed a linear decrease of the CD_225nm_ intensity during their melting, indicating the absence of a triple helix structure (Figure S4). When the pH was adjusted from neutral to either acidic or basic, the favorable electrostatic interaction between Asp and Lys became much weakened, resulting in the loss of the capability to initiate the formation of heterotrimer. It indicated that pH played a dominant role in modulating the stability of the heterotrimer AAB, confirming the MD simulation results that favorable salt bridges were responsible for triggering the heterotrimer formation.

## 3. Discussion

The various chain composition of heterogeneous proteins plays a determinative role in modulating their physiological and pathological processes [2,3,4]. Type I collagen, the most important component of various connective tissues, is composed of two identical α1 chains and one α2 chain, representing a typical heterotrimeric protein [10,11]. Heterotrimeric type I collagen regulates diverse cellular processes and interactions with extracellular matrix molecules, while its homotrimeric isoform has been demonstrated to relate to fibrosis, tumors, and other diseases [17]. Due to its complex native environment and huge supramolecular size, it is very difficult to study the property based on natural type I collagen [26,32]. Smaller synthetic peptides have been developed to decipher the structure and function of type I collagen [21,22]. However, these studies frequently depend on the traditional single-domain peptides consisting of repeating (Gly-X-Y)n, resulting in the lack of versatility to incorporate novel functional motifs.

We have herein developed a set of triblock peptides that can assemble into stable collagen mimetic heterotrimers with desired functional sequences and free from the interference of its compositional homotrimers. The triblock peptides, consisting of a central collagen-like block and two oppositely charged N-/C-terminal blocks, showed intrinsically monomer conformation due to the electrostatic repulsion between the same charged amino acids. In contrast, the mixture of two paired triblock peptides with complementary terminal charged sequences resulted in the formation of a stable heterotrimer due to the electrostatic attraction. The thermal stability of 2A:1B mixtures at pH 3.0 and 11.0 further demonstrated the pivotal role of electrostatic interaction in regulating the formation of heterotrimers. Upon adjusting the pH from neutral to either acidic or basic conditions, the favorable electrostatic interaction between the charged amino acids, Asp and Lys, significantly attenuated, leading to the inability to form heterotrimers [33,34].

It is noteworthy that desired amino acid sequences such as functional motifs can be conveniently introduced into the central block without interference in the heterotrimer formation. CD characterization of the reconstituted peptides, K-α1-D and D-α2-K as well as K-α2-D and D-α1-K, demonstrated the successful construction of heterotrimers mimicking the two different chains α1 and α2 in type I collagen. The independence of the collagen-like block from the two terminal blocks enables the triblock peptide system with versatility that the current single-domain heterotrimer model peptides lack [35,36]. NMR results indicated that the 2A*:1B* mixture led to the formation of A*A*B* heterotrimer composed of two A chains and one B chain; while the 1A*:2B* mixture resulted in the formation of A*B*B* heterotrimer comprising two A chains and one B chain, suggesting that the heterotrimer composition precisely regulated by the relative concentration of the two triblock peptides [32]. MD simulations suggested the formation of a salt-bridge network between the oppositely charged amino acids in the paired triblock peptides, which has been demonstrated to contribute to the formation of stable heterotrimers [20,24,26]. These triblock peptides provide a robust and versatile strategy to mimic the structure and composition of heterotrimeric collagen. which have promising applications in the fields of tissue. Furthermore, the triblock peptides provide a potential strategy to create self-assembled heterotrimeric peptide-based biomaterials that can recapitulate the high-order structure and function of collagen and decipher collagen-related diseases, which have promising applications in the fields of tissue engineering and regenerative medicine.

## 4. Materials and Methods

### 4.1. Peptide Synthesis

All peptides were synthesized in-house by the standard Fmoc solid phase synthesis method using rink amide resin at a 0.1 mmol scale, and the Fmoc protection group of resin was removed with 20% piperidine in DMF. Briefly, stepwise couplings of amino acids were performed using a double coupling method with Fmoc amino acids (4 eq.), DIEA (6 eq.), and activator reagents (HBTU + HOBT 0.66 mmol/mL, 4 eq.). The reaction mixture was washed with DMF (3 × 5 mL) and DCM (3 × 5 mL) following each step of coupling, and the Fmoc protection group was removed with 20% piperidine in DMF. The test reagent (2% ethanal DMF, 2% chloranil DMF) was employed to monitor the completion of each coupling reaction and Fmoc deprotection. The peptides were obtained by treating the resin with TFA/TIS/H_2_O (95:2.5:2.5) for 3 h to remove the tBu groups and to cleave itself from the resin. Cold Et_2_O was used to precipitate the peptide. The precipitates were resuspended in cold Et_2_O and centrifuged again. The crude products were then dissolved in water and lyophilized. The peptides were purified by GL Biochem (Shanghai, China) Ltd., using reverse phase HPLC on a Gemini-NX 5μm C18 110A column (4.6 × 250 mm) (Figures S5–S10). The identity of the peptides was confirmed by mass spectrometry (MS) (Figures S11–S18).

### 4.2. Circular Dichroism Spectroscopy

Circular dichroism (CD) experiments were performed on a Chirascan Instrument (Applied Photophysics Ltd., Leatherhead, UK) using optically matched quartz cuvettes with a path length of 1.0 mm (Model 110-OS, Hellma, Plainview, NY, USA). Thermal unfolding curves were measured by monitoring the amplitude of the characteristic CD band at 225 nm, while the temperature was increased by 1 °C per step from 4 °C to 60 °C at a heating rate of 10 °C/h. For homotrimers, peptide solutions with a concentration of 0.45 mM in a 10 mM phosphate buffer (pH 7.0) were used for all the experiments. For heterotrimers, various peptides were mixed at desired ratios in such a way that the final total peptide concentration was 0.45 mM in 10 mM phosphate buffer (pH 7.0). In addition, peptides K-α1-D and D-α2-K were mixed with a ratio of 2:1 at pH 3.0 and 11.0, respectively, and the final total peptide concentration was 0.45 mM. Thermal unfolding studies were performed with and without preheating. For preheating studies, peptides were mixed at desired ratios, heated to 85 °C, and incubated for 20 min. The peptide solution was slowly cooled to room temperature and then incubated at 4 °C for 24 h before performing the unfolding studies. For non-preheating studies, peptides were mixed at a ratio of 1:1, and the unfolding studies were performed immediately. The minimum of the derivative of the fraction folded plot is used in this paper to indicate the melting temperature (T_m_) under the conditions described above.

### 4.3. Solution-State Nuclear Magnetic Resonance Spectroscopy

Nuclear magnetic resonance (NMR) experiments were performed using a 700-MHz Bruker Avance III spectrometer (Bruker, MA, USA) equipped with a cryogenic triple-resonance TXI probe (Bruker, MA, USA). NMR samples were prepared in 10% D2O/90% PBS (10 mM, pH 7.0) at a total concentration of 9 mM. The samples were incubated at 85 °C for 20 min, slowly cooled to room temperature, and then incubated at 4 °C for 24 h to allow the complete formation of a triple helix. Then, 2D 1H-15N heteronuclear single quantum coherence (HSQC) experiments were acquired at 8 °C. For measurements of amide proton temperature gradients, a series of 1H-15N HSQC spectra were recorded at various temperatures from 8 °C to 23 °C with an interval of 3 °C. Amide proton temperature gradients were obtained by linear regression analysis of the amide proton chemical shift versus temperature. All the NMR data were processed using the Topspin program.

### 4.4. Molecular Dynamics Simulation

The Triple-Helical Collagen Building Script was used to create the fully atomistic model of the selected wild-type collagen sequence with GPO triplets using THe BuScr. The three-dimensional structure of triple-helical collagen molecule was predicted by THeBuScr1 (http://structbio.biochem.dal.ca/jrainey/THeBuScr.html (accessed on 22 November 2022)) [37]. The initial coordinates of peptide AAB were constructed. The Tleap module of the Amber 20.0 program with the Amberff19SB force field was used for the preliminary treatment of peptides. The structure was immersed into a cubic periodic box of TIP3P waters with water molecules. The distance from the surface of the box to the closest atom of the solute was set to 12 Å in periodic boundary conditions to avoid the boundary effect. All the molecular dynamics (MD) simulations were performed using the Amber 20.0 program. First, energy minimization was carried out by steepest descent and conjugated gradient methods to remove the bad contacts between the solute and solvent. Then, the systems were gradually heated from 0 to 300.0 K in the NVT ensemble with the protein constrained by 10 kcal mol^−1^ Å^−2^. After the heating, the constraint of peptides was gradually reduced, and five-step equilibrium simulations were performed (50 ps per step) in the NPT ensemble. One nanosecond equilibration was followed by 600 ns MD simulations without any restraint in the NPT ensemble at a temperature of 310.0 K and a pressure of 1 atm. The Particle Mesh Ewald (PME) method was used to calculate long-range electrostatics interactions [38], and the nonbonded cutoff distance was set to 10 Å. The SHAKE algorithm was applied to restrain all covalent bonds containing hydrogen atoms. Amber was used to compute the root mean square deviation (RMSD) and radius of gyration (Rg). Salt-bridge analysis was performed using Discovery Studio 4.5 Client software [26]. The salt bridges within a distance of 6.0 Å were selected for this study.

## Figures and Tables

**Table 1:** Rational design of triblock heterotrimer peptides. ^15^N-labeled glycine was marked with an asterisk.

Peptide Name	Peptide Sequence	T_m_/°C
α1	(GPO)_3_GFOGER(GPO)_3_	38.0
α2	(GPO)_3_GPOGES(GPO)_3_	41.0
K-α1-D	K_5_(GPO)_3_GFOGER(GPO)_3_D_5_	<4.0
D-α1-K	D_5_(GPO)_3_GFOGER(GPO)_3_K_5_	<4.0
K-α2-D	K_5_(GPO)_3_GPOGES(GPO)_3_D_5_	<4.0
D-α2-K	D_5_(GPO)_3_GPOGES(GPO)_3_K_5_	<4.0
K-α1*-D	K_5_(GPO)_3_GFOG*ER(GPO)_3_D_5_	-
D-α2*-K	D_5_(GPO)_3_GPOG*ES(GPO)_3_K_5_	-

## Data Availability

The data supporting the findings of this study are presented in this article including the Supplementary Materials and are available from the corresponding author upon reasonable request.

## Supplementary Materials

The supporting information can be downloaded at: https://www.mdpi.com/article/10.3390/ijms25126550/s1.
