# Supplementary material for: Versatile Self-Assembly of Triblock Peptides into Stable Collagen Mimetic Heterotrimers
Source: Int J Mol Sci. 2024 Jun 14;25(12):6550. doi: 10.3390/ijms25126550 (PMC11203499; doi:10.3390/ijms25126550)
Supplement: Supplementary file 1 [file ijms-25-06550-s001.zip › ijms-2986794-supplementary.pdf]

Supporting Information

Versatile Self-Assembly of Triblock Peptides into Stable Collagen Mimetic Heterotrimers

Linyan Yao[1,2], Biyang Ling[1], Sha Zhao[3], Fansen Yu[4], Huanxiang Liu[4], Shenlin Wang[3], and Jianxi Xiao[1,*]

1 State Key Laboratory of Applied Organic Chemistry, College of Chemistry and Chemical Engineering, Lan-zhou University, Lanzhou 730000, China

2 School of Life Science, Lanzhou University, Lanzhou 730000, China

3 College of Chemistry and Molecular Engineering, Beijing NMR Center, Peking University, Beijing, 100871, China

4 School of Pharmacy, Lanzhou University, Lanzhou 730000, China

* Correspondence: xiaojx@lzu.edu.cn

Table S1. The melting temperature of paired triblock heterotrimer peptides at different ratios. Peptide mixtures at ratios of 2:1, 1:2 and 1:1 were prepared with preheating. The peptide mixtures at a 1:1 ratio without preheating were marked with an asterisk.

**Peptide/Peptide mixture**	**Ratio**	**T_m/°C**
K-α1-D/D-α1-K	2:1	29.0
	1:2	31.0
	1:1	30.0
	1:1*	31.0
K-α2-D/D-α2-K	2:1	30.0
	1:2	31.0
	1:1	30.0
	1:1*	30.0

Table S2. The melting temperature of triblock heterotrimer peptides mimicking the natural composition of type I collagen at different ratios.

**Peptide/Peptide mixture**	**Ratio**	**T_m/°C**
K-α1-D/D-α2-K	2:1	31.0
	1:2	29.0
	1:1	31.0
D-α1-K/K-α2-D	2:1	32.0
	1:2	31.0
	1:1	30.0

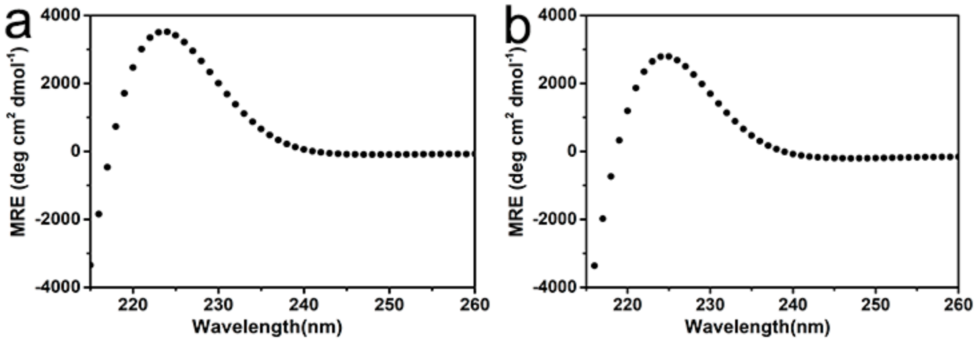

Figure S1. CD spectra of peptides α1 (a) and α2 (b) at 4 °C in 10 mM phosphate buffer (pH 7.0).

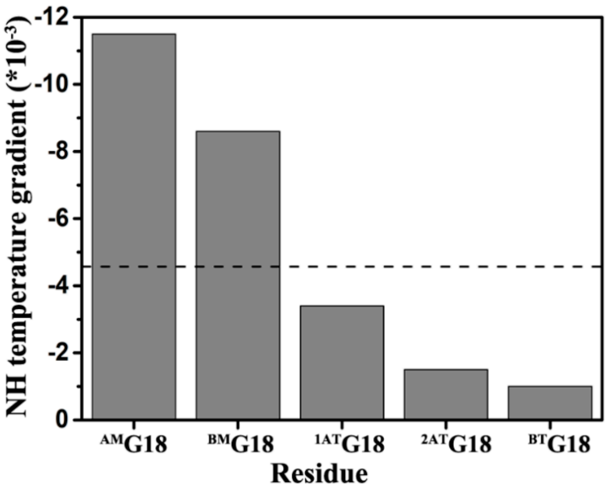

Figure S2. Amide proton NH temperature gradients of peptide 2A*:1B*. The black dashed horizontal line corresponds to a cut-off value for hydrogen bonding, with less negative values than −4.6 ppb/°C indicative of hydrogen bonding.

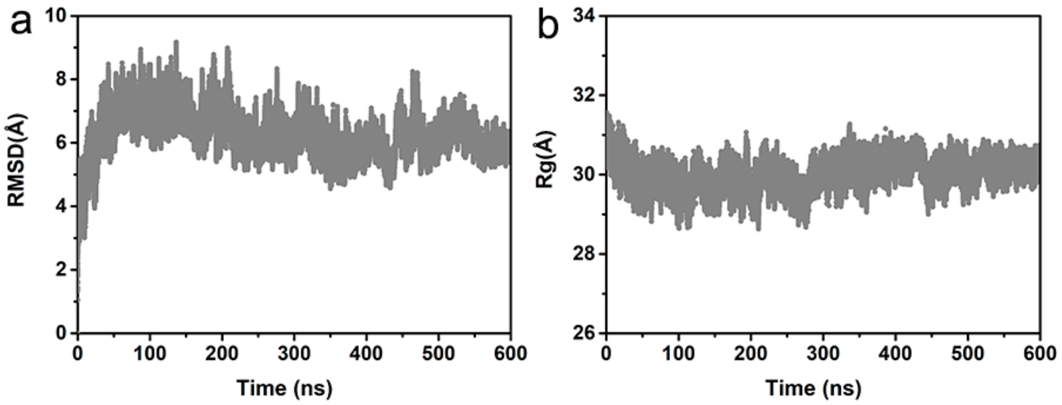

Figure S3. Graphs depicting RMSD (a) and Rg of the heterotrimer peptide model AAB (b).

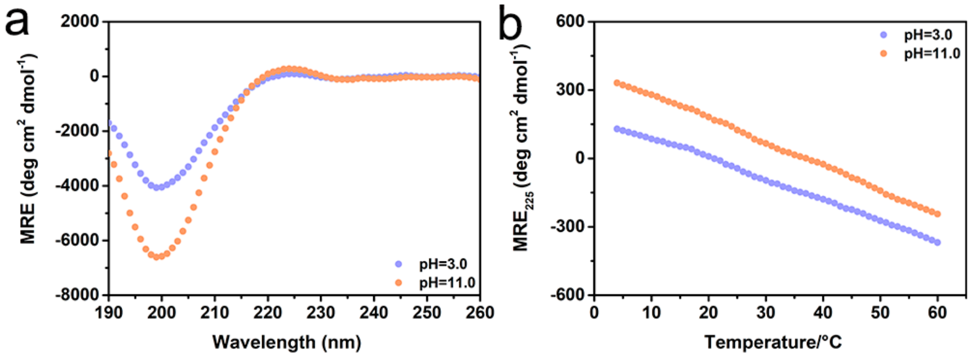

Figure S4. CD characterization of the mixtures of peptides K-α1-D and D-α2-K with a ratio of 2:1 at different pHs: 3.0 (purple) and 11.0 (orange). CD spectra (a) and CD thermal unfolding (b).

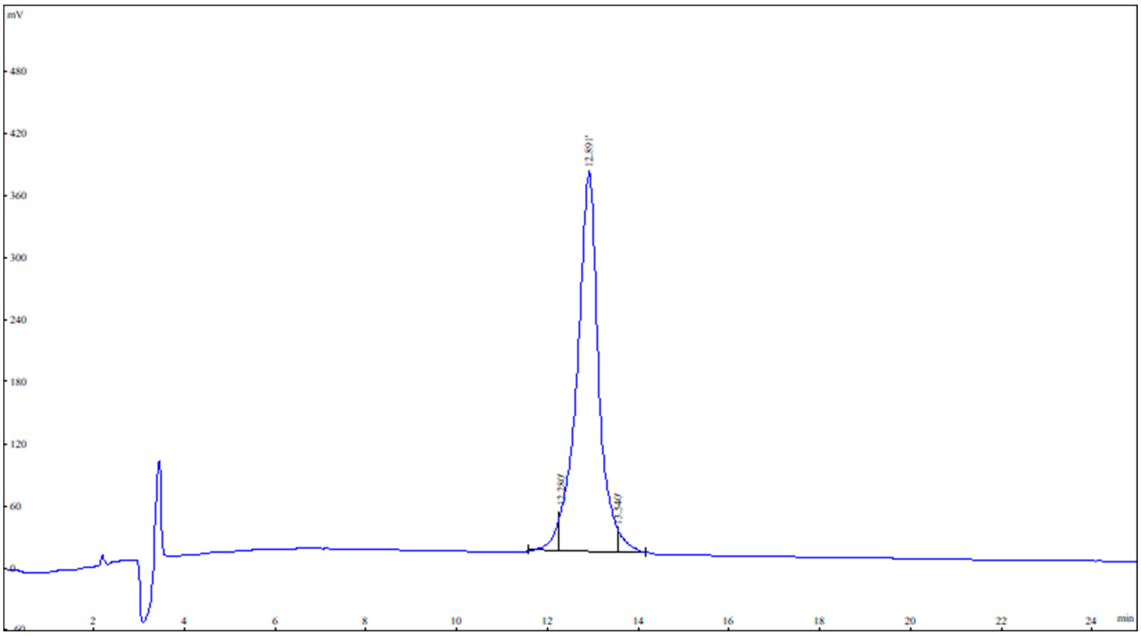

Figure S5. HPLC characterization of peptide K-α1-D.

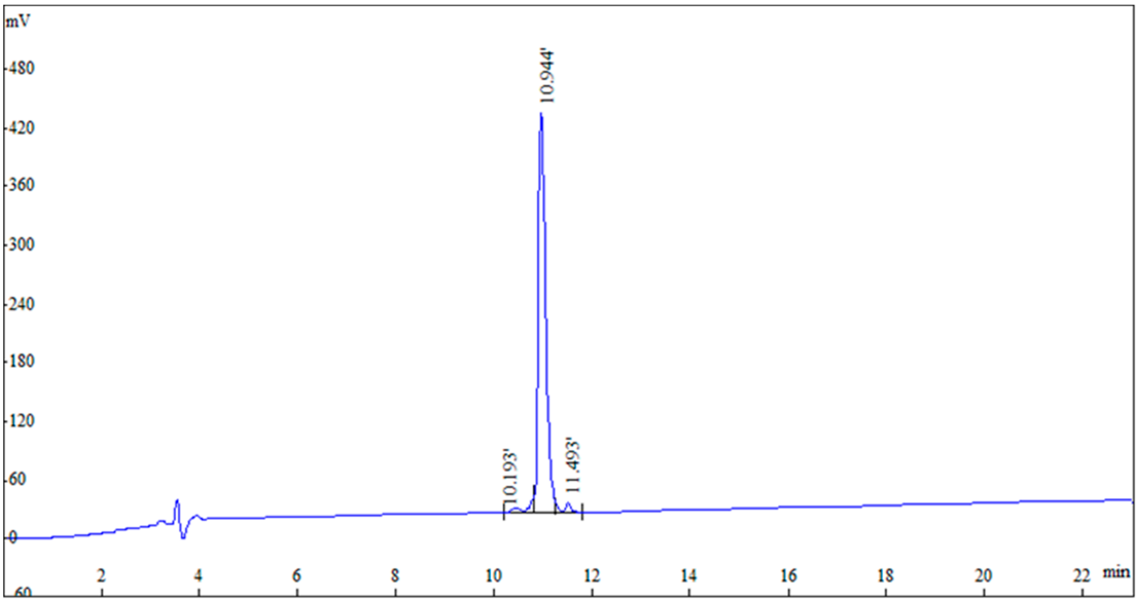

Figure S6. HPLC characterization of peptide D-α1-K.

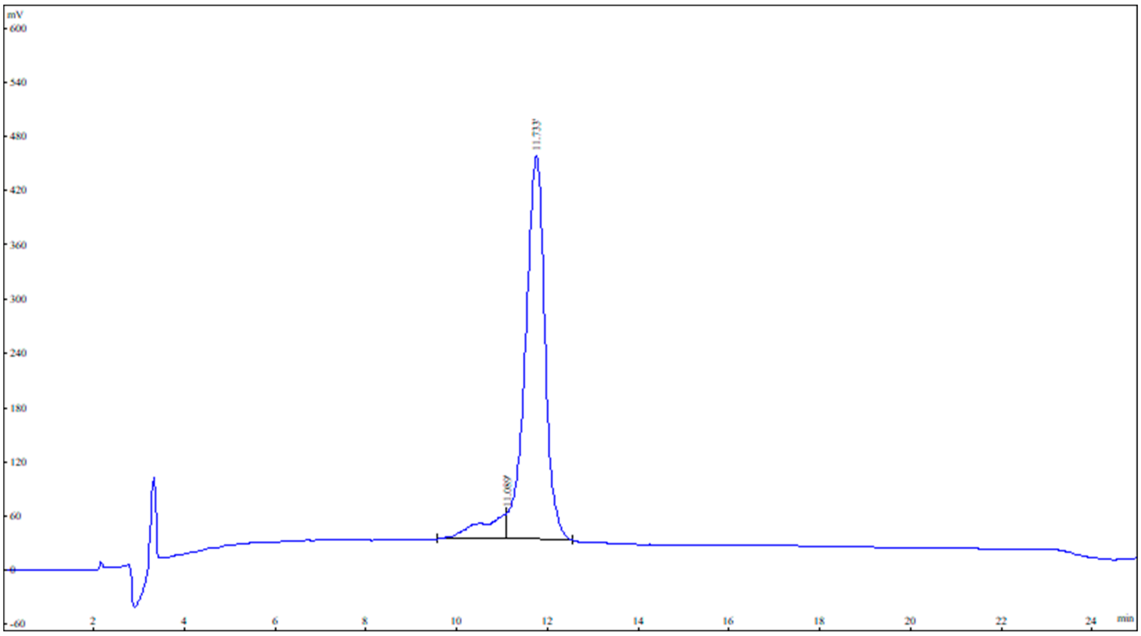

Figure S7. HPLC characterization of peptide K-α2-D.

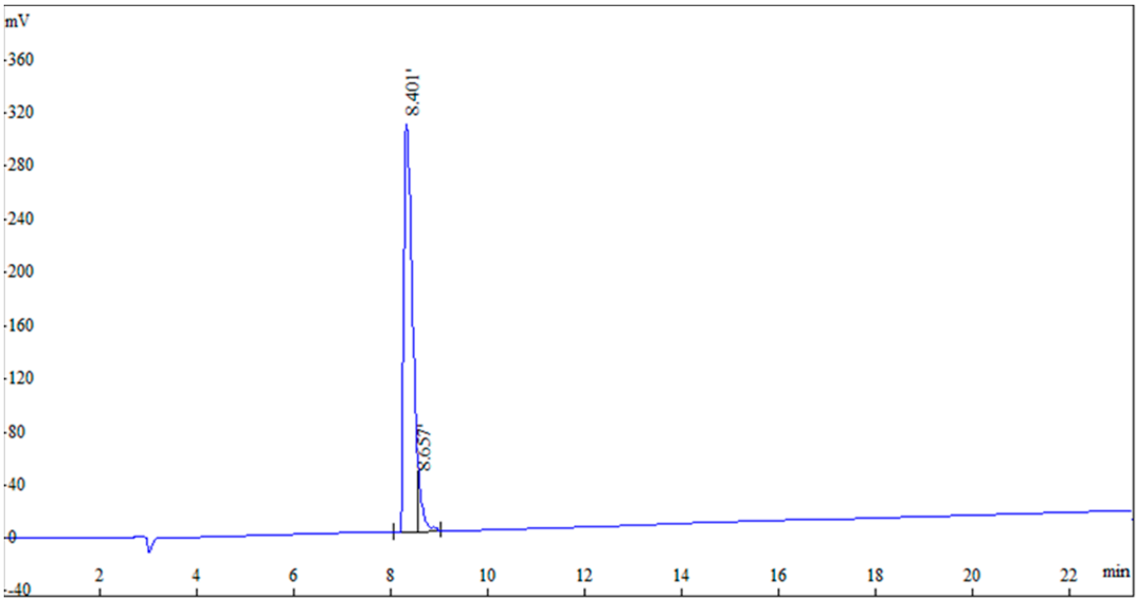

Figure S8. HPLC characterization of peptide D-α2-K.

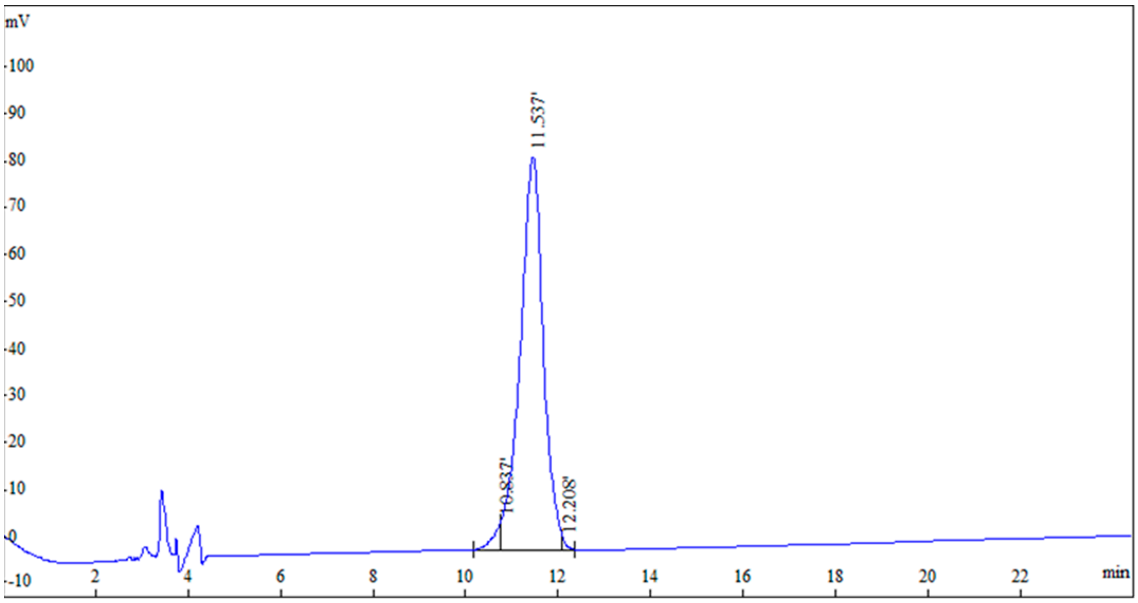

Figure S9. HPLC characterization of peptide K-α1*-D.

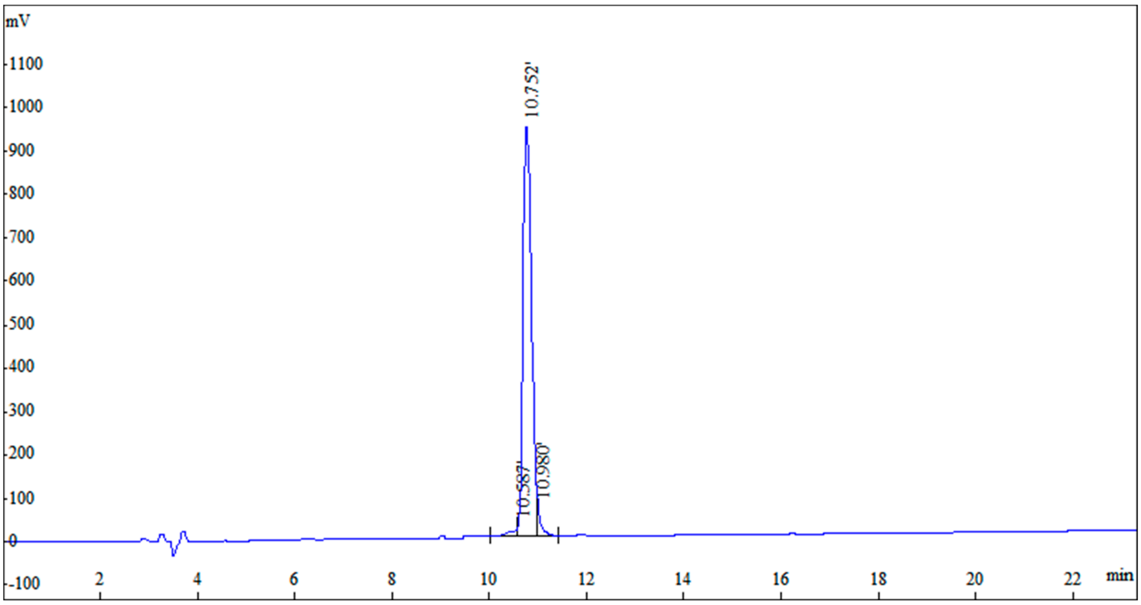

Figure S10. HPLC characterization of peptide D-α2*-K.

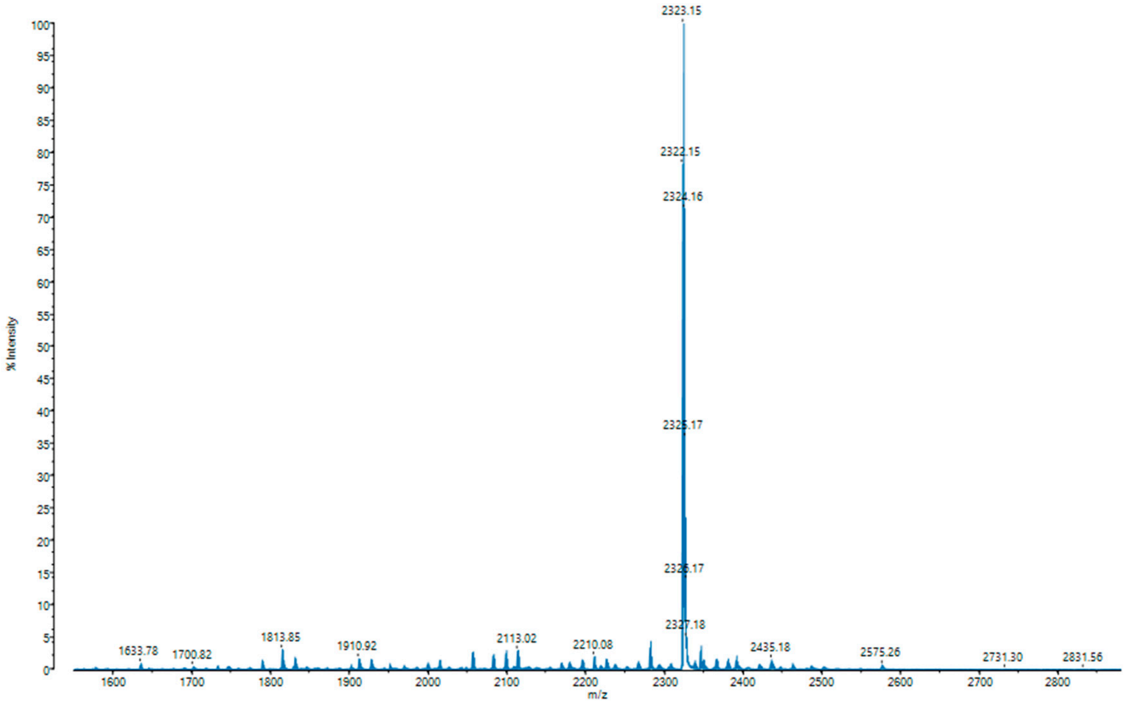

Figure S11. MS characterization of peptide α1.

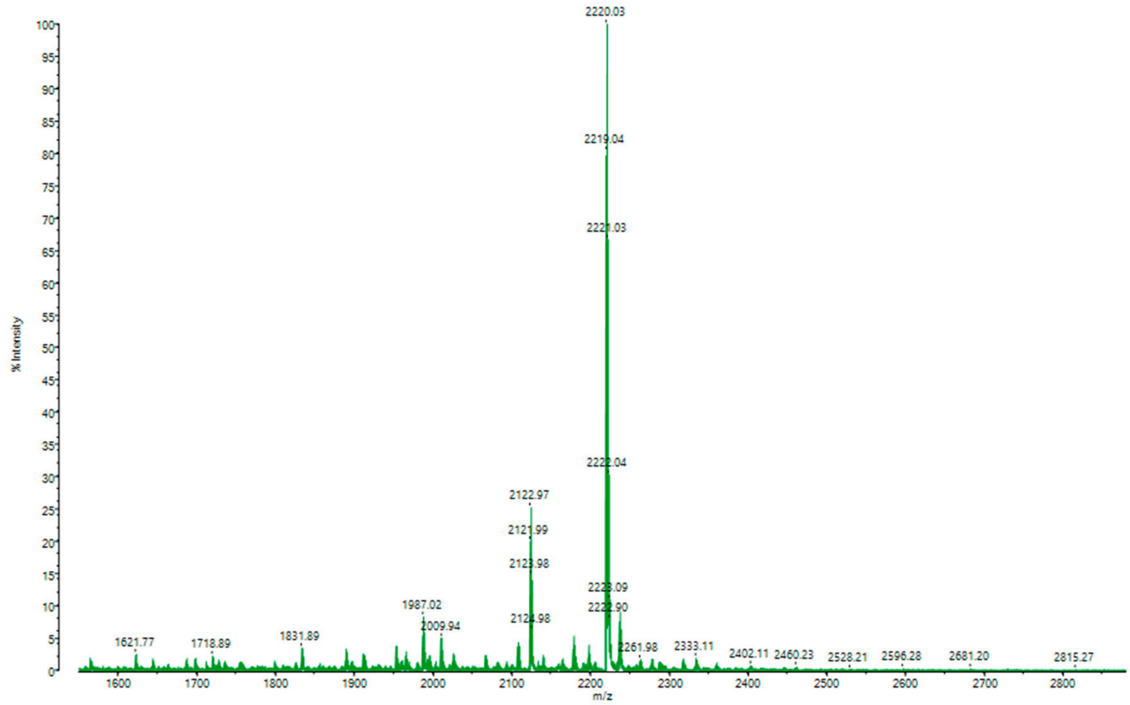

Figure S12. MS characterization of peptide α2.

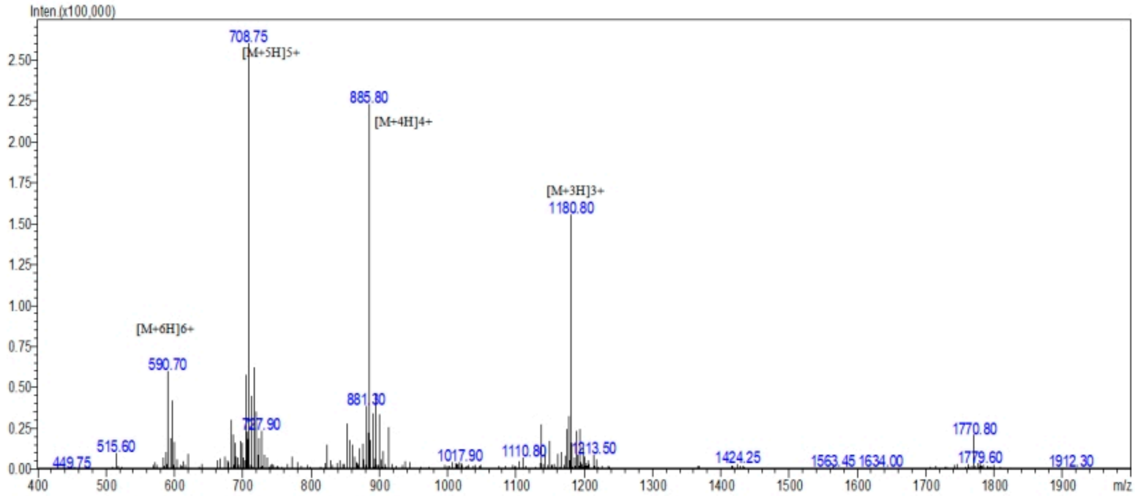

Figure S13. MS characterization of peptide K-α1-D.

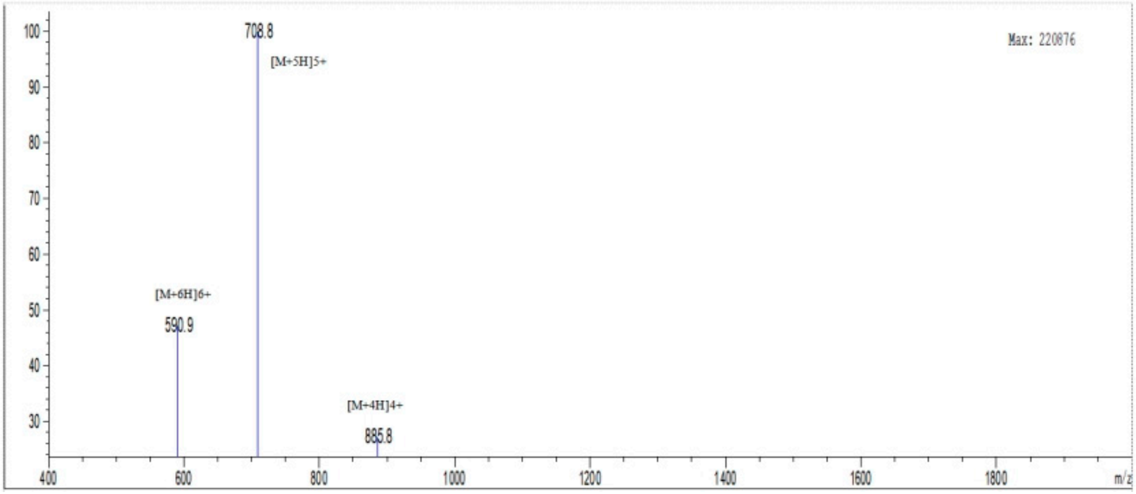

Figure S14. MS characterization of peptide D-α1-K.

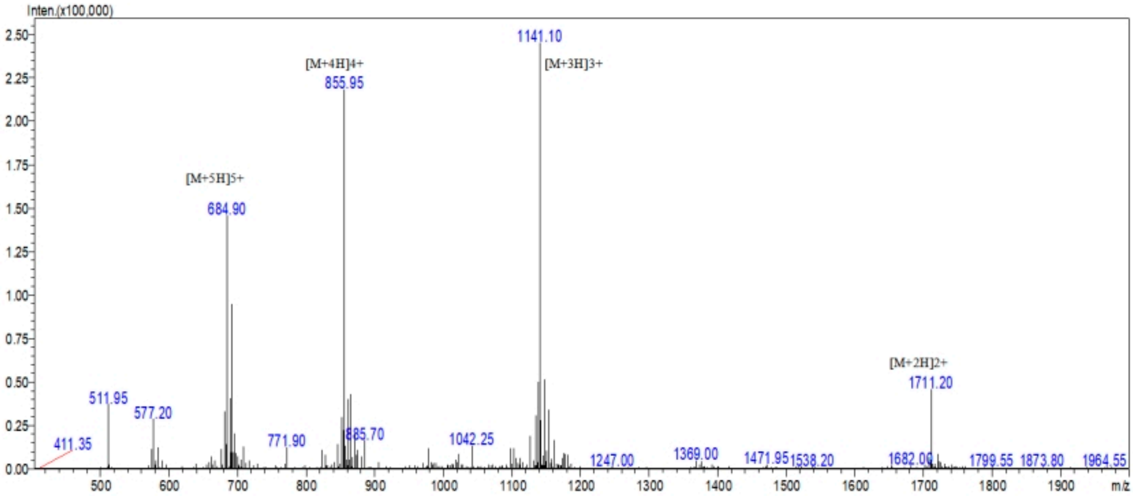

Figure S15. MS characterization of peptide K-α2-D.

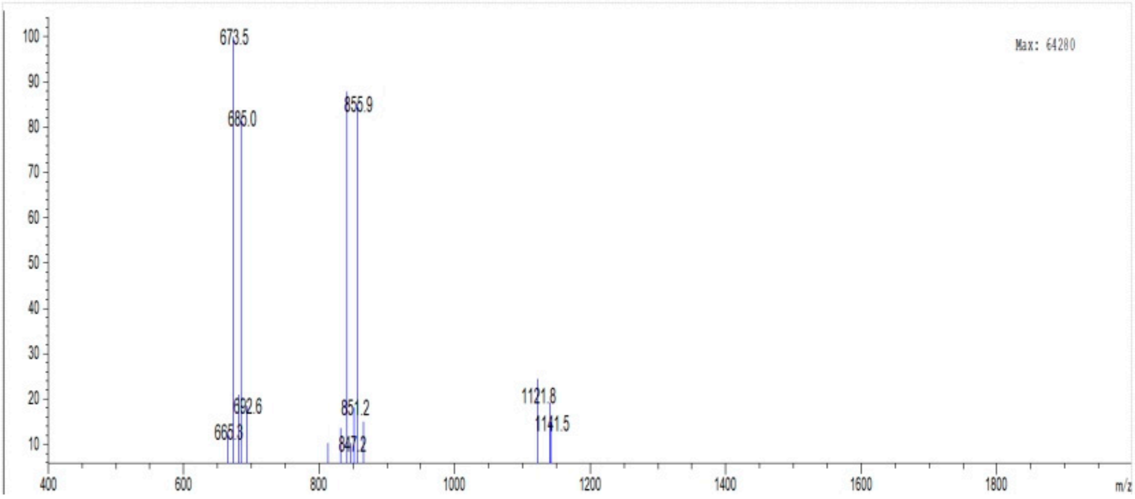

Figure S16. MS characterization of peptide D-α2-K.

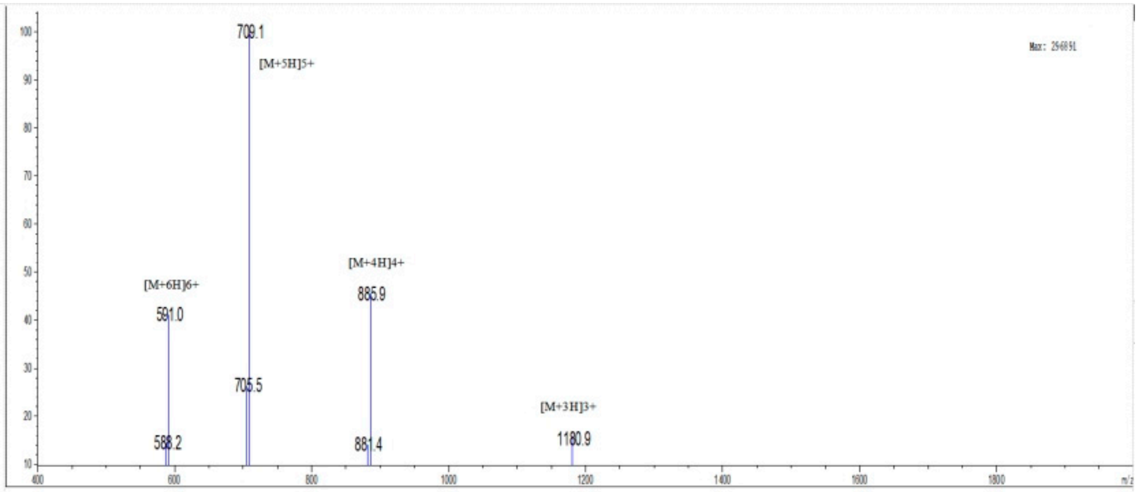

Figure S17. MS characterization of peptide K-α1*-D.

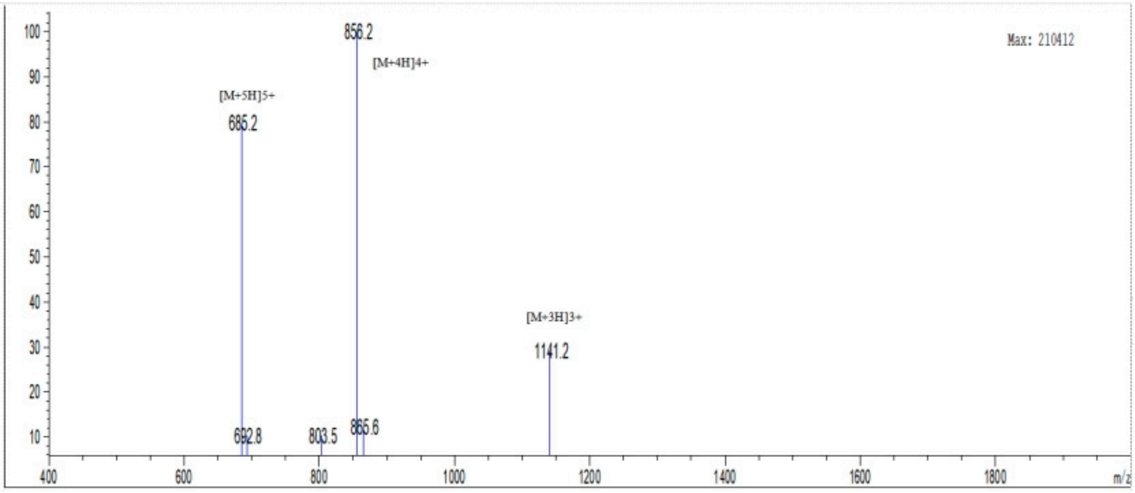

Figure S18. MS characterization of peptide D-α2*-K.
